# Association of Cognitive Function Trajectories in Centenarians With Postmortem Neuropathology, Physical Health, and Other Risk Factors for Cognitive Decline

**DOI:** 10.1001/jamanetworkopen.2020.31654

**Published:** 2021-01-15

**Authors:** Nina Beker, Andrea Ganz, Marc Hulsman, Thomas Klausch, Ben A. Schmand, Philip Scheltens, Sietske A. M. Sikkes, Henne Holstege

**Affiliations:** 1 Alzheimer Center Amsterdam, Department of Neurology, Amsterdam Neuroscience, Vrije Universiteit Amsterdam, Amsterdam UMC, Amsterdam, the Netherlands; 2 Center for Neurogenomics and Cognitive Research, Department of Molecular and Cellular Neuroscience, Amsterdam Neuroscience, Vrije Universiteit Amsterdam, Amsterdam, the Netherlands; 3 Department of Pathology, Amsterdam Neuroscience, Amsterdam UMC, Amsterdam, the Netherlands; 4 Department of Clinical Genetics, Amsterdam Neuroscience, Vrije Universiteit Amsterdam, Amsterdam UMC, Amsterdam, the Netherlands; 5 Amsterdam Public Health Research Institute, Department of Epidemiology and Biostatistics, Amsterdam UMC, Vrije Universiteit Amsterdam, Amsterdam, the Netherlands; 6 Brain & Cognition, Department of Psychology, University of Amsterdam, Amsterdam, the Netherlands; 7 Department of Clinical Psychology, Neuropsychology and Developmental Psychology, Faculty of Behavioural and Movement Sciences, Vrije Universiteit Amsterdam, Amsterdam, the Netherlands

## Abstract

**Importance:**

Understanding mechanisms associated with prolonged cognitive health in combination with exceptional longevity might lead to approaches to enable successful aging.

**Objective:**

To investigate trajectories of cognitive functioning in centenarians across domains, and to examine the association of these trajectories with factors underlying cognitive reserve, physical health, and postmortem levels of Alzheimer disease (AD)–associated neuropathology.

**Design, Setting, and Participants:**

This cohort study used neuropsychological test data and postmortem neuropathological reports from Dutch centenarians who were drawn from the 100-plus Study between January 2013 and April 2019. Eligible participants self-reported being cognitively healthy, which was confirmed by a proxy. Data analysis was performed between June 2019 and June 2020.

**Exposures:**

Age, sex, *APOE* ε genotype, factors of cognitive reserve, physical health, and AD-associated neuropathology (ie, amyloid-β, neurofibrillary tangles, and neuritic plaques).

**Main Outcomes and Measures:**

In annual visits (until death or until participation was no longer possible), centenarians underwent an extensive neuropsychological test battery, from which an mean *z* score of global cognition, memory, executive functions, verbal fluency, visuospatial functions, and attention/processing speed was calculated. Linear mixed models with a random intercept and time as independent variable were used to investigate cognitive trajectories, adjusted for sex, age, education, and vision and hearing capacities. In a second step, linear mixed models were used to associate cognitive trajectories with factors underlying cognitive reserve, physical health at baseline, and AD-associated neuropathology.

**Results:**

Of the 1023 centenarians approached, 340 were included in the study. We analyzed 330 centenarians for whom cognitive tests were available at baseline (239 [72.4%] women; median [interquartile range] age of 100.5 [100.2-101.7] years), with a mean (SD) follow-up duration of 1.6 (0.8) years. We observed no decline across investigated cognitive domains, with the exception of a slight decline in memory function (β, −0.10 SD per year; 95% CI, −0.14 to −0.05 SD; *P* < .001). Cognitive performance was associated with factors of physical health (eg, higher Barthel index: β, 0.37 SD per year; 95% CI, 0.24-0.49; *P* < .001) and cognitive reserve (eg, higher education: β, 0.41 SD per year; 95% CI, 0.29-0.53; *P* < .001), but none of these factors were associated with the rate of decline. Neuropathological reports were available for 44 participants. While centenarian brains revealed varying loads of postmortem neuropathological hallmarks of AD, this was not associated with cognitive performance or rate of decline.

**Conclusions and Relevance:**

While we observed a slight vulnerability for decline in memory function, centenarians maintained high levels of performance in all other investigated cognitive domains for up to 4 years despite the presence of risk factors of cognitive decline. These findings suggest that mechanisms of resilience may underlie the prolongation of cognitive health until exceptional ages.

## Key Points

**Question:**

Are cognitively healthy centenarians resilient against further cognitive decline?

**Findings:**

In this cohort study of 330 self-reported cognitively healthy centenarians, cognitive trajectories revealed only a slight decline in memory functioning, while other domains remained stable over time. Centenarians maintained high levels of cognitive performance despite being exposed to varying levels of risk factors of cognitive decline, including postmortem Alzheimer disease–associated neuropathologies.

**Meaning:**

These findings indicate that prolonged maintenance of cognitive functioning may be supported by mechanisms underlying resilience against risk factors of cognitive decline.

## Teaser

This cohort study examines trajectories of cognitive function and their associations with cognitive reserve, physical health, and postmortem levels of neuropathology associated with Alzheimer disease in Dutch centenarians.

## Introduction

Some individuals reach ages beyond 100 years and become centenarians with intact cognitive functions,^1,2,3,4,5^ which indicates that cognitive impairment is not inevitable at extreme ages. Cross-sectional and longitudinal studies in younger age groups (20-90 years) have shown that aging is accompanied by a maintenance in language, semantic knowledge, abstract reasoning, and visuospatial functions, whereas a vulnerability is observed in domains such as processing speed, executive functions, and episodic and working memory.^6,7,8,9,10,11^ It is still unclear to what extent individuals who maintain cognitive health until age 100 years escape or delay decline across different cognitive domains. Based on the 40% incidence of dementia at age 100 years, and assuming a continued increase beyond 100, it is to be expected that a decline in cognitive functions will be observable in this age group.^12,13^

In this study, we aim to identify trajectories of cognitive performance in different domains for cognitively healthy centenarians, and to explore associations with risk factors of cognitive decline, including neuropathology associated with Alzheimer disease (AD) and factors of cognitive reserve.^14,15^

## Methods

### Participants

The 100-plus Study is a prospective cohort study of centenarians who self-report to be cognitively healthy, as confirmed by a relative or caregiver. Trained researchers visited the centenarians at their homes annually to subject them to questionnaires regarding demographic characteristics, medical history, and measurements of physical functions and cognitive testing. The Medical Ethics Committee of the Amsterdam UMC approved this study and informed consent was obtained from all participants. Brain donors signed informed consent for brain donation. The study was conducted in accordance with the Declaration of Helsinki.^16^ Detailed participant recruitment and procedures for the study were described previously.^17^ This study followed the Strengthening the Reporting of Observational Studies in Epidemiology (STROBE) reporting guideline.

Between January 2013 and April 2019, we approached 1023 centenarians, of whom 340 were included in the study. Included in the analyses of the current study are 330 centenarians who completed at least 1 of the neuropsychological tests at baseline. Neuropathological assessment was available for 44 centenarians. We reported follow-up data for up to 4 years (see eFigure 1 in the Supplement for a flow chart on participation duration). Data were analyzed between June 2019 and June 2020.

### Assessment of Cognitive Functioning

Scores on the Mini-Mental State Examination (MMSE) were used to describe baseline cognitive performance.^18^ For in-depth investigation of cognitive trajectories we calculated mean *z* scores for memory, executive functions, verbal fluency, visuospatial functions, and attention/processing speed combined based on neuropsychological test scores (see eAppendix in the Supplement). Missing test scores were imputed using multiple imputation with chained equations^19^ based on predictive mean matching method (see eAppendix in the Supplement). Global cognition was investigated using a composite score across all domains. Test administration and implemented adaptations were described previously.^20^ Memory was evaluated using the story recall subtest of the Rivermead Behavioral Memory Test and the Visual Association Test A.^21,22^ Executive functions were evaluated using the Trail Making Test (TMT) B (scores were reversed, such that higher scores indicate better performance), key search subtest of the Behavioral Assessment of the Dysexecutive Syndrome Test Battery, and the digit span backward subtest of the Wechsler Adult Intelligence Scale (WAIS-III).^23,24,25^ Verbal fluency was measured using the Controlled Oral Word Association Test (Letter fluency, D-A-T) and animal fluency.^26,27^ Visuospatial functions were evaluated with the number location subtest of the Visual Object and Space Perception Battery^28^ and the clock drawing test.^29,30^ Attention/processing speed were evaluated with the digit span forward subtest of the WAIS-III and the TMT A.^23,25^

### Assessment of Risk Factors

#### Demographic Factors

In addition to sex and age, this study also considered *APOE* status as a risk factor. Carrying 1 or 2 *APOE* ε4 alleles was considered risk increasing, carrying 1 or 2 *APOE* ε2 alleles was considered protective, and *APOE* ε2ε4 was considered risk increasing.^17,31,32,33,34^

#### Physical Health

The Barthel Index evaluates independence in performing activities of daily living.^35^ Grip strength was determined in kg with a hand dynamometer (JAMAR) in duplicate in the left and right hand.^36^ Hearing and vision were annotated based on our observations and by the centenarian and proxy report.^17,20^ Living situation was classified as living independently or living dependently (ie, care center facility, with family or when 24/7 care help available). History of stroke and/or transient ischemic attack and hypertension was determined based on the GP report or centenarian and proxy report.

#### Cognitive Reserve

Factors considered in assessing cognitive reserve included education level (International Standard Classification of Education, 1997 Revision),^37^ cognitive activity questionnaire filled in together with family members (assessment of frequency of cognitive activity in the past [age 6 until age 40 years] and currently),^38^ and premorbid intelligence (Dutch Adult Reading Test).^39,40,41^ Missing items on questionnaires were imputed (see eAppendix in the Supplement).

#### Neuropathological Hallmarks

Autopsies were performed in collaboration with the Netherlands Brain Bank.^42^ We assessed the load of amyloid-β (Aβ) in extracellular plaques using Thal phase,^43^ the level of intracellular accumulation of phosphorylated tau protein in neurofibrillary tangles (NFTs) using Braak stages,^44,45,46^ and the load of neuritic plaques (NPs; a subtype of plaques containing dystrophic neurites) according to Consortium to Establish a Registry for Alzheimers Disease (CERAD) scores.^47^

### Statistical Analyses

Cognitive trajectories were estimated using linear mixed models (LMMs), with each domain as dependent variable (including a random intercept for each participant) and time (ie, the duration of the follow up in years after inclusion) as independent continuous variable. LMMs with time as a dummy variable were also performed to estimate trajectories per year separately. To explore the variability in the trajectories, we investigated whether a random slope should be included in the LMMs and whether we could distinguish centenarians whose functioning remained stable from centenarians who declined by using latent class linear mixed models (LCLMMs). According to the lowest bayesian information criterion, we evaluated whether the LCLMMs with 1 or 2 classes showed the best fit.^48,49^

LMMs were also performed to explore associations with risk factors (measured at baseline) on levels of cognitive scores aggregated over the study period. Parameters were estimated by restricted maximum likelihood.^50^ Next, we estimated the interaction between risk factors measured at baseline with the rate of cognitive change for domains that significantly declined (time × factor).

All models were adjusted for sex, age, education, and hearing and vision abilities at time of testing (all these factors were mean-centered). Verbal fluency was not adjusted for vision ability, as these tests seemed independent or were found independent of vision ability.^20^ Despite the explorative nature of this study, we conservatively corrected for multiple testing using Benjamini-Hochberg procedure to decrease the risk of reporting chance discoveries. Two-sided *P* values < .05 after correction for multiple comparisons were considered significant. Statistical analyses were performed with *R* version 3.5.2 with lme4 and hlme packages (R Project for Statistical Computing).

## Results

### Baseline Characteristics and Postmortem Neuropathology

Of the 330 centenarians, 239 (72.4%) were women, and the group had a median (interquartile range [IQR]) age of 100.5 (100.2–101.7) years. The median (IQR) education level was 3 (1-4) on the international standard scale (ie, upper secondary education). A total of 187 (56.7%) of the centenarians lived independently, and the majority had good vision and hearing capacities (211 [65.5%] and 184 [56.4%] participants, respectively). We observed varying levels of neuropathology in the brains of the 44 donors (median [IQR] Thal phase, 3 [1–4]; Braak stage, 3 [3–4]; and CERAD score, 1 [0–1]). While all Thal phases were present, the highest Braak stage (6) and highest CERAD score (3) were absent. See Table 1 for an overview of all characteristics.

**Table 1.** Baseline Demographic and Clinical Characteristics

Characteristics	Participants, No. (%)^a^
Age, median (IQR), y	100.5 (100.2-101.7)
Women	239 (72.4)
*APOE* ε4 allele carriers	48 (16.8)
ε4ε4/ε3ε4/ε2ε4	1/33/14
*APOE* ε3 homozygous	173 (60.5)
*APOE* ε2 allele carriers	65 (22.7)
ε2ε2/ε2ε3	2/63
Independent living situation	187 (56.7)
Mobility	
Able to walk independently^b^	245 (78.8)
Able to walk with help of another person	17 (5.5)
Able to move independently in a wheelchair	25 (8.0)
Not able to move independently in a wheelchair	24 (7.7)
Vision	
Good	211 (65.5)
Moderate	43 (13.4)
Poor	36 (11.2)
Very poor	32 (9.9)
Hearing	
Good	184 (56.4)
Moderate	105 (32.2)
Poor	32 (9.8)
Very poor	5 (1.5)
MMSE score, median (IQR)	25.2 (22.0-27.6)
Barthel Index, independent in ADL	172 (57.3)
Prior stroke or transient ischemic attack	92 (30.3)
Hypertension	191 (63.0)
Grip strength, mean (SD), kg	15.5 (6.2)
Education, median (IQR), ISCED level	3 (1-4)
Highest ISCED education level	125 (37.9)
Premorbid IQ, mean (SD)	98.2 (14.3)
Cognitive activity, mean (SD)	
Lifetime	47.1 (13.5)
Current	13.0 (4.2)
Amyloid-β (Thal phase), median (IQR)	3.0 (1.0-4.0)
Neurofibrillary tangles (Braak stage), median (IQR)	3.0 (3.0-4.0)
Neuritic plaques (CERAD score), median (IQR)	1.0 (0-1.0)

Abbreviations: ADL, activities of daily living; CERAD, Consortium to Establish a Registry for Alzheimer Disease; IQR, interquartile range; ISCED, International Standard Classification of Education; MMSE, Mini-Mental State Examination.

^a^ There were missing data on mobility (19 participants), vision (8 participants), hearing (4 participants), Barthel Index (30 participants), stroke or transient ischemic attack (26 participants), hypertension (27 participants), grip strength (169 participants), *APOE* ε allele (44 participants), premorbid IQ (90 participants), lifetime cognitive activity (38 participants), and current cognitive activity (43 participants). Neuropathological data were available for a subgroup of 44 (9 men, 35 women) centenarians. Score ranges are as follows: ISCED education level (0-6, with the highest level ≥postsecondary nontertiary education), Barthel Index (0-20, scores ≥15 indicating independence in ADL), grip strength was measured in kg (the maximum score out of 4 attempts was used as the final score), lifetime and current cognitive activity (score range 0-100, and 0-25 respectively, higher scores indicate more frequent cognitive activity), MMSE (score range 0-30, with higher scores indicating better performance), Thal phase (score range 0-5), Braak stage (score range 0-6), and CERAD score (score range 0-3); higher scores indicate higher levels of pathology.

^b^ With or without help of a walking stick or walker.

### Cognitive Trajectories

We applied LMMs to estimate cognitive trajectories for each domain separately. The duration of follow-up ranged from 0 to 4 years, with a mean (SD) follow-up duration of 1.6 (0.8) years (for mean test scores at each time point, see eTable 1 in the Supplement).

**Figure 1.**
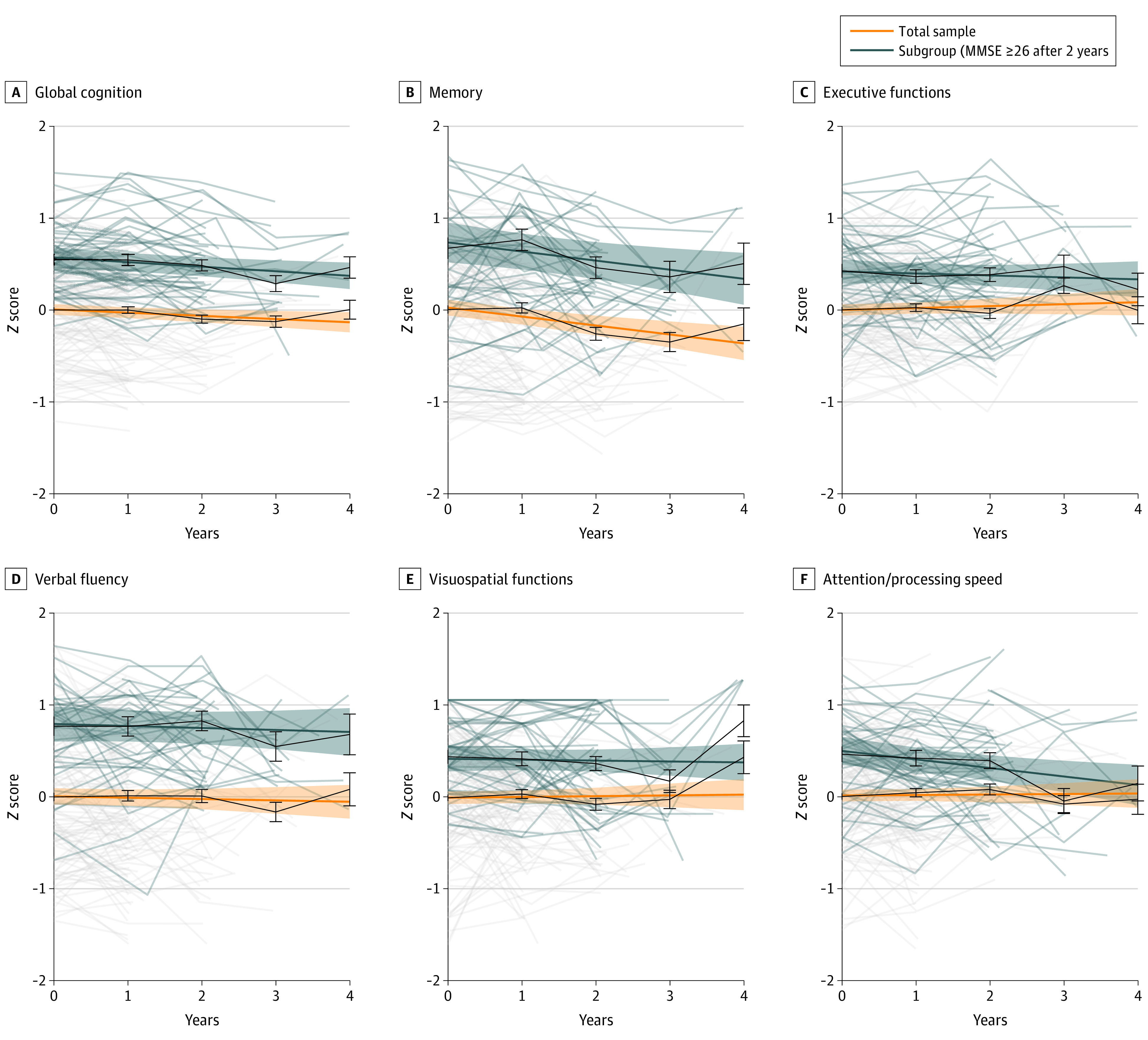
Mean and Individual Trajectories of Cognitive Domains Trajectories of cognitive function are based on linear mixed models with random intercept, adjusted for sex, age, education, and hearing and vision capacities. The model on verbal fluency was only adjusted for hearing capacities. Pale lines indicate individual trajectories (raw scores), darker colored lines indicate mean trajectories with time as a continuous variable, and black lines indicate mean trajectories with time as a dummy variable.

LMMs with a random intercept indicated no significant change over the time after study inclusion in the performance in executive functions, verbal fluency, visuospatial functions, and attention/processing speed (Figure 1, Table 2). We observed a mean 0.10 SD decline (95% CI, −0.14 to −0.05 SD; *P* < .001) in memory performance. Global cognition declined 0.03 SD per year (95% CI, −0.06 to −0.01 SD; *P* = .01). When memory tests were excluded from the global domain score, the changes were not significant (β, −0.01; 95% CI, −0.04 to 0.02; *P* = .43). This suggests that the significant difference in global cognitive functioning is driven by the difference in memory scores. Moreover, we observed that memory functioning increasingly declined with a longer follow-up duration (eFigure 2 in the Supplement).

**Table 2.** Regression Coefficients From Linear Mixed Models to Investigate Change Over Time in Cognitive Domains^a^

Model	Global cognition		Memory		Executive functions		Verbal fluency		Visuospatial functions		Attention/processing speed	
	β (95% CI)	*P* value	β (95% CI)	*P* value	β (95% CI)	*P* value	β (95% CI)	*P* value	β (95% CI)	*P* value	β (95% CI)	*P* value
**Total sample (n = 330)**												
Linear time model												
Time	−0.03 (−0.06 to −0.01)	.01^b^	−0.10 (−0.14 to −0.05)	<.001^b^	0.02 (−0.02 to 0.06)	.28	−0.01 (−0.06 to 0.03)	.53	0.01 (−0.04 to 0.05)	.72	0.01 (−0.04 to 0.05)	.79
Discrete time model												
Year 1	−0.00 (−0.06 to 0.05)	.97	0.02 (−0.08 to 0.11)	.72	0.02 (−0.05 to 0.10)	.55	0.01 (−0.08 to 0.11)	.79	0.04 (−0.05 to 0.14)	.39	0.04 (−0.05 to 0.13)	.36
Year 2	−0.10 (−0.17 to −0.03)	.008^b^	−0.26 (−0.39 to −0.14)	<.001^b^	−0.04 (−0.14 to 0.07)	.47	0.01 (−0.12 to 0.14)	.87	−0.07 (−0.20 to 0.06)	.27	0.08 (−0.04 to 0.19)	.20
Year 3	−0.13 (−0.24 to −0.01)	.03	−0.35 (−0.55 to −0.16)	<.001^b^	0.26 (0.10 to 0.43)	.002^b^	−0.16 (−0.36 to 0.04)	.11	−0.02 (−0.21 to 0.18)	.87	−0.08 (−0.26 to 0.10)	.37
Year 4	0.00 (−0.19 to 0.20)	.97	−0.16 (−0.50 to 0.18)	.37	−0.00 (−0.29 to 0.28)	.98	0.08 (−0.26 to 0.43)	.64	0.44 (0.09 to 0.79)	.01	−0.03 (−0.35 to 0.29)	.85
**MMSE ≥26 after 2 y (n = 43)**												
Linear time model												
Time	−0.05 (−0.09 to −0.01)	.01^b^	−0.10 (−0.17 to −0.02)	.01	−0.02 (−0.08 to 0.04)	.49	−0.02 (−0.09 to 0.05)	.56	−0.01 (−0.07 to 0.05)	.76	−0.09 (−0.16 to −0.03)	.006^a^
Discrete time model												
Year 1	−0.01 (−0.11 to 0.09)	.92	0.09 (−0.11 to 0.29)	.37	−0.06 (−0.23 to 0.10)	.46	−0.00 (−0.20 to 0.19)	.98	−0.02 (−0.18 to 0.14)	.79	−0.04 (−0.22 to 0.13)	.63
Year 2	−0.06 (−0.17 to 0.04)	.22	−0.21 (−0.42 to −0.01)	.04	−0.04 (−0.21 to 0.13)	.63	0.06 (−0.14 to 0.26)	.57	−0.07 (−0.24 to 0.09)	.38	−0.07 (−0.25 to 0.11)	.45
Year 3	−0.26 (−0.42 to −0.10)	.002^b^	−0.31 (−0.63 to 0.00)	.06	0.05 (−0.21 to 0.31)	.73	−0.22 (−0.53 to 0.09)	.17	−0.27 (−0.52 to −0.01)	.04	−0.51 (−0.78 to −0.24)	<.001^a^
Year 4	−0.09 (−0.31 to 0.13)	.44	−0.17 (−0.60 to 0.27)	.45	−0.20 (−0.56 to 0.15)	.27	−0.09 (−0.52 to 0.34)	.68	0.39 (0.05 to 0.74)	.03	−0.32 (−0.69 to 0.06)	.10

Abbreviation: MMSE, Mini-Mental State Examination.

^a^ Linear time models are defined as linear models with time as a continuous variable for each cognitive domain separately. Discrete time models are separate linear models with time as a dummy variable for each cognitive domain to investigate the trajectories for every year separately. All models include a random intercept, and were adjusted for sex, age, education, and vision and hearing capacities. The models on verbal fluency were only adjusted for hearing capacities. A random slope was not included.

^b^ Significant after correction for multiple testing (listed *P* values are uncorrected for multiple comparisons).

Results were similar when we applied LMMs to investigate the trajectories for every year separately (Table 2). Furthermore, since only a few observations were available for each centenarian, we could not include random slopes in the LMMs,^51^ such that we could not estimate the variability of the rates of decline between individual trajectories after study inclusion or during a specific year after study inclusion.

To further allow exploration of the presence of separate clusters within the cognitive trajectories, we fitted LCLMMs for models that included 1 and 2 classes. For all cognitive domains, the 1-class model had a better fit (lowest bayesian information criterion values) as opposed to the 2-class model (eTable 2 in the Supplement). This indicates that we did not identify a subset of centenarians with a differential trajectory of cognitive decline.

LMMs were also repeated for a subgroup of 43 centenarians who were previously identified to survive longest and who maintained a high level of global cognitive health (MMSE score ≥26) for at least 2 years after study inclusion.^52^ We observed that, during the follow-up period, their level of performance on all domains was higher compared with the total sample of centenarians (Figure 1). They showed a slight decline of a mean 0.10 SD in memory performance (95% CI, −0.17 to −0.02 SD; *P* = .01), although this was not significant after correction for multiple comparisons (Table 2). Moreover, they declined a mean 0.09 SD in attention/processing speed of which the effect appeared in the third year of follow-up (95% CI, −0.16 to −0.03 SD; *P* = .006).

### Associations Between Risk Factors and Cognitive Trajectories

LMMs were performed to explore (1) the association of risk factors measured at baseline with levels of cognitive performance aggregated over the study period (Table 3) and (2) to explore interactions between risk factors measured at baseline and the rate of decline in global cognition and memory respectively (eTable 3 in the Supplement).

**Table 3.** Linear Mixed Model Regression Coefficients to Investigate the Association of Risk Factors With Levels of Cognitive Performance Aggregated Over the Study Period^a^

Models	Global cognition		Memory		Executive functions		Verbal fluency		Visuospatial functions		Attention/processing speed	
	β (95% CI)	*P* value	β (95% CI)	*P* value	β (95% CI)	*P* value	β (95% CI)	*P* value	β (95% CI)	*P* value	β (95% CI)	*P* value
Age	−0.02 (−0.06 to 0.02)	.26	−0.03 (−0.08 to 0.03)	.33	−0.04 (−0.08 to 0.00)	.06	−0.02 (−0.08 to 0.04)	.52	−0.01 (−0.06 to 0.03)	.53	0 (−0.05 to 0.04)	.84
Men	0.05 (−0.08 to 0.18)	.43	0.10 (−0.09 to 0.29)	.30	0.06 (−0.07 to 0.19)	.38	−0.13 (−0.33 to 0.07)	.21	0.10 (−0.04 to 0.25)	.17	0.13 (−0.01 to 0.27)	.07
APOE ε4	−0.11 (−0.27 to 0.05)	.19	−0.26 (−0.51 to −0.02)	.04	−0.07 (−0.23 to 0.09)	.38	−0.08 (−0.32 to 0.17)	.53	0.04 (−0.15 to 0.22)	.71	−0.14 (−0.31 to 0.03)	.12
APOE ε2	0.09 (−0.05 to 0.24)	.21	0.16 (−0.05 to 0.38)	.14	0.08 (−0.06 to 0.22)	.26	0.10 (−0.12 to 0.31)	.38	0.11 (−0.05 to 0.27)	.19	−0.01 (−0.16 to 0.14)	.88
Factors of physical health												
Independent living situation	0.21 (0.09 to 0.32)	.001^b^	0.26 (0.09 to 0.43)	.003^b^	0.13 (0.01 to 0.24)	.04	0.33 (0.16 to 0.51)	<.001^b^	0.15 (0.02 to 0.28)	.02	0.17 (0.04 to 0.29)	.008^b^
Good hearing	0.13 (0.01 to 0.25)	.03	0.09 (−0.08 to 0.26)	.30	0.06 (−0.06 to 0.18)	.31	0.16 (−0.02 to 0.34)	.08	0.12 (−0.01 to 0.25)	.08	0.18 (0.06 to 0.31)	.005^b^
Good vision	0.20 (0.08 to 0.33)	.001^b^	0.13 (−0.05 to 0.30)	.17	0.21 (0.08 to 0.33)	.001^b^	0.12 (−0.07 to 0.30)	.21	0.15 (0.02 to 0.29)	.03	0.38 (0.24 to 0.51)	<.001^b^
Barthel Index, ADL independence	0.37 (0.24 to 0.49)	<.001^b^	0.39 (0.20 to 0.57)	<.001^b^	0.27 (0.14 to 0.40)	<.001^b^	0.55 (0.36 to 0.73)	<.001^b^	0.35 (0.20 to 0.49)	<.001^b^	0.25 (0.11 to 0.38)	.001^b^
Previous stroke or TIA	−0.12 (−0.25 to 0.01)	.07	−0.16 (−0.35 to 0.03)	.10	−0.09 (−0.22 to 0.04)	.18	−0.20 (−0.40 to −0.00)	.05	−0.04 (−0.18 to 0.11)	.62	−0.07 (−0.20 to 0.07)	.35
Hypertension	0.05 (−0.08 to 0.17)	.48	0.05 (−0.13 to 0.23)	.58	0.04 (−0.08 to 0.17)	.49	0.05 (−0.14 to 0.24)	.64	0.08 (−0.06 to 0.22)	.25	0.06 (−0.08 to 0.19)	.40
Grip strength	0.02 (0.00 to 0.03)	.04	0.02 (0.00 to 0.04)	.03	0.01 (−0.00 to 0.03)	.16	0.03 (0.00 to 0.05)	.03	0.02 (−0.00 to 0.03)	.07	−0.00 (−0.02 to 0.01)	.67
Factors of cognitive reserve												
≥Postsecondary, nontertiary education	0.41 (0.29 to 0.53)	<.001^b^	0.31 (0.14 to 0.48)	<.001^b^	0.47 (0.35 to 0.59)	<.001^b^	0.46 (0.28 to 0.64)	<.001^b^	0.26 (0.12 to 0.39)	<.001^b^	0.50 (0.37 to 0.63)	<.001^b^
Premorbid IQ	0.02 (0.01 to 0.02)	<.001^b^	0.02 (0.01 to 0.03)	<.001^b^	0.02 (0.01 to 0.02)	<.001^b^	0.02 (0.01 to 0.03)	<.001^b^	0.02 (0.01 to 0.02)	<.001^b^	0.02 (0.01 to 0.02)	<.001^b^
Lifetime cognitive activity	0.01 (0.00 to 0.01)	.008^b^	0.01 (0.00 to 0.02)	.02	0.00 (−0.00 to 0.01)	.14	0.01 (−0.00 to 0.01)	.07	0.01 (0.00 to 0.01)	.02	0.01 (0.00 to 0.01)	.008^b^
Current cognitive activity	0.04 (0.02 to 0.06)	<.001^b^	0.04 (0.02 to 0.07)	.001^b^	0.03 (0.01 to 0.05)	.001^b^	0.06 (0.04 to 0.08)	<.001^b^	0.03 (0.01 to 0.05)	.003^b^	0.04 (0.02 to 0.05)	<.001^b^
AD-associated neuropathologies												
Aß (Thal phase)	−0.05 (−0.15 to 0.06)	.40	−0.09 (−0.26 to 0.09)	.35	−0.09 (−0.21 to 0.04)	.18	0.02 (−0.15 to 0.19)	.84	−0.07 (−0.20 to 0.05)	.27	−0.02 (−0.12 to 0.08)	.68
NFTs (Braak stage)	−0.17 (−0.38 to 0.03)	.10	−0.22 (−0.56 to 0.11)	.20	−0.22 (−0.45 to 0.02)	.08	−0.01 (−0.35 to 0.32)	.93	−0.29 (−0.52 to −0.06)	.02	−0.12 (−0.31 to 0.08)	.25
NPs												
CERAD score 1	−0.05 (−0.42 to 0.31)	.77	−0.00 (−0.60 to 0.59)	.99	−0.14 (−0.56 to 0.27)	.51	0.14 (−0.45 to 0.72)	.65	−0.24 (−0.67 to 0.18)	.27	−0.07 (−0.42 to 0.29)	.72
CERAD score 2	−0.22 (−0.62 to 0.18)	.30	−0.22 (−0.88 to 0.43)	.51	−0.36 (−0.81 to 0.10)	.14	−0.00 (−0.64 to 0.64)	.99	−0.32 (−0.78 to 0.14)	.18	−0.19 (−0.56 to 0.19)	.34

Abbreviations: AD, Alzheimer disease; ADL, activities of daily living; Aβ, amyloid-β; CERAD, Consortium to Establish a Registry for Alzheimer disease; NFTs, neurofibrillary tangles; NPs, neuritic plaques; TIA, transient ischemic attack.

^a^ Separate linear mixed models including a random intercept adjusted for sex, age, education, and vision and hearing capacities. Models on verbal fluency were only adjusted for hearing capacities. A random slope was not included. There were missing data on *APOE* ε allele (44 participants), vision (8 participants), hearing (4 participants), Barthel Index (30 participants), stroke or TIA (26 participants), hypertension (27 participants), grip strength (169 participants), premorbid IQ (90 participants), lifetime cognitive activity (38 participants), and current cognitive activity (43 participants). Score ranges are as follows: Barthel Index (0-20, scores ≥15 indicating independence in ADL), lifetime and current cognitive activity (score range 0-100 and 0-25, respectively; higher scores indicate more frequent cognitive activity), Thal phase (score range 0-5), Braak stage (score range 0-6), and CERAD score (score range 0-3) (higher scores indicate higher levels of pathology for these 3 measures).

^b^ Significant after correction for multiple testing (listed *P* values are uncorrected for multiple comparisons).

#### Aggregated Models

Centenarians who lived independently at baseline performed significantly better than those who lived dependently on all domains except executive functions (global cognition: β, 0.21 SD per year; 95% CI, 0.09 to 0.32 SD; *P* = .001). A higher baseline Barthel Index score was associated with higher performance on all domains (global cognition: β, 0.37; 95% CI, 0.24 to 0.49; *P* < .001). Good hearing was associated with higher performance on attention/processing speed (β, 0.18; 95% CI, 0.06 to 0.31; *P* = .005), and good vision was associated with higher performance on global cognition (β, 0.20; 95% CI, 0.08 to 0.33; *P* = .001), executive functions, and attention/processing speed, but not with other cognitive domains after correction for multiple comparisons.

Higher levels of education (global cognition: β, 0.41; 95% CI, 0.29 to 0.53; *P* < .001), premorbid IQ (global cognition: β, 0.02; 95% CI, 0.01 to 0.02; *P* < .001), and frequency of current cognitive activity (global cognition: β, 0.04; 95% CI, 0.02 to 0.06; *P* < .001) were associated with higher scores on all domains. Lifetime cognitive activity was associated with better performance on global cognition (β, 0.01; 95% CI, 0.00 to 0.01; *P* = .008) and attention/processing speed (β, 0.01; 95% CI, 0.00 to 0.01; *P* = .001). None of the other investigated associations between risk factors with performance on cognitive domains were significant after correction for multiple comparisons (Table 3).

#### Interaction Effects Models

**Figure 2.**
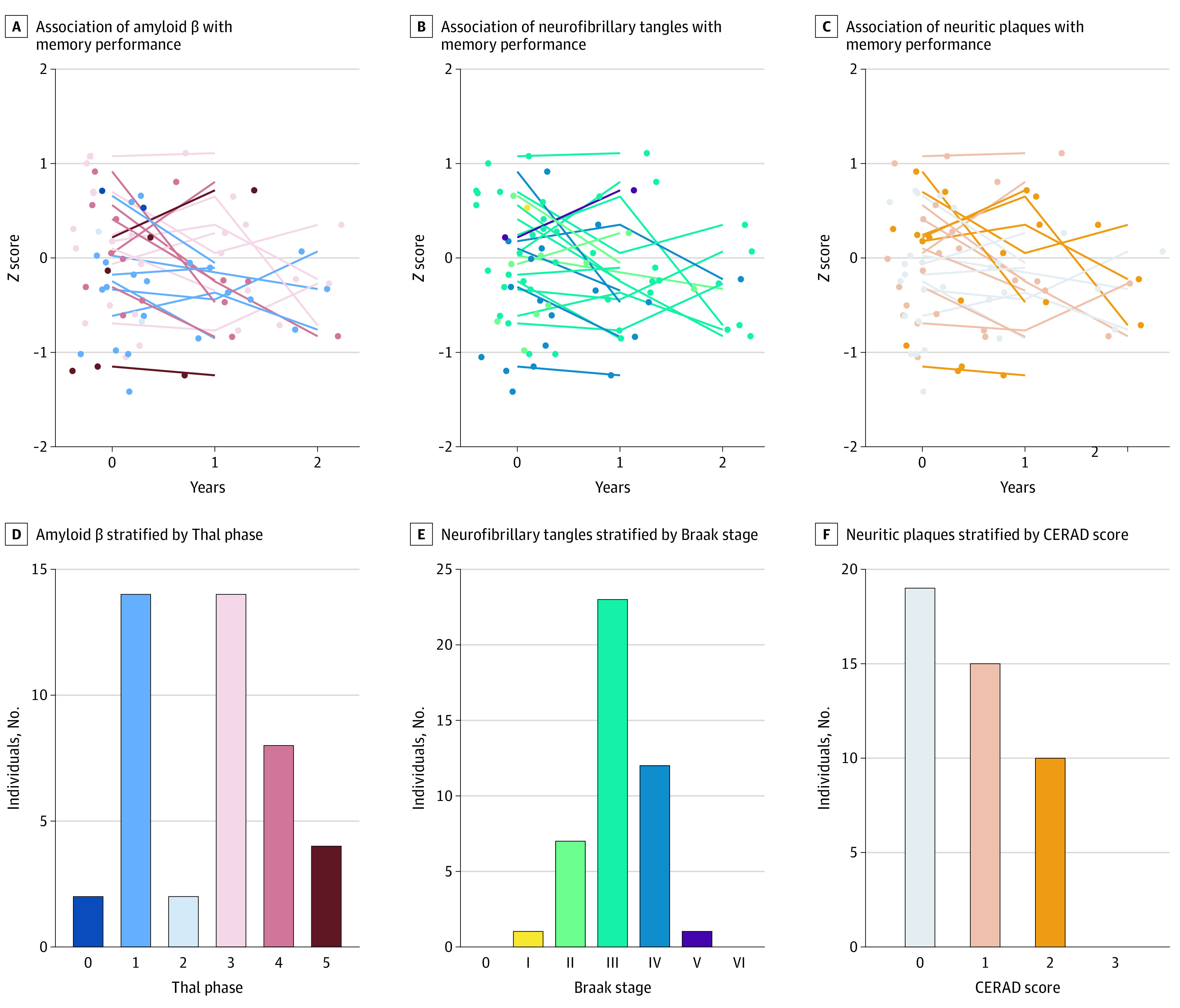
Trajectories of Memory Performance Colors of lines and dots match with neuropathological scores of the same color in the subfigures below them. *Z* scores on memory per time point for amyloid-β (Thal phases), neurofibrillary tangles (Braak stages), and neuritic plaques (CERAD scores).

Since we observed a significant but slight decline in global cognition and memory, we tested whether there were interactions between risk factors measured at baseline and the rate of decline in these 2 domains. We found that only good hearing was associated with a higher rate of decline on global cognition (β, −0.07; 95% CI, −0.12 to −0.02; *P* = .01 with *P* < .05 after correction for multiple testing). None of the other investigated interactions were significant after correction for multiple comparisons (eTable 3 in the Supplement), including the varying loads of Aβ, NFTs, and NPs (Figure 2).

## Discussion

The centenarians in our cohort maintained their levels of performance in most cognitive domains for up to 4 years. We observed a slight decline in global cognition, which was driven by a decline in memory function. Factors underlying cognitive reserve and physical health were associated with levels of cognitive performance, but not with the decline in function thereafter. Postmortem findings revealed varying loads of Aβ (assessed using Thal phases), NFTs (using Braak stages), and NPs (using CERAD scores), none of which were associated with cognitive performance or decline.

Dementia risk increases exponentially with age and reaches approximately 40% per year for people aged 100 years old.^12,13^ This exponential increase implies that a person who lives between 70 and 95 years is exposed to the same dementia risk as a person who lives between age 100 and 102: an estimated 60%.^13,17^ Thus, 25 years of dementia risk in the younger population is compressed into 2 years in centenarians. This indicates that prolonged stability of cognitive functioning in centenarians may be considered more extraordinary than in nonagenarians. Indeed, we had expected to observe more evidence of cognitive decline than only a minor decrement in memory function. Nevertheless, the observed memory decline of 0.10 SD per year in cognitively healthy centenarians is higher compared with the memory decline of 0.03 SD per year observed in cognitively healthy community dwellers aged 65 to 85 years.^11^ But the decline is lower compared with the decline observed in AD patients aged approximately 65 years, whose memory function declined 0.90 SD per year,^53^ which suggests that the observed decline in our cohort may not be AD-based.

When we focused on the highest performing centenarians within this cohort (those who scored ≥26 on the MMSE during several years after study inclusion),^52^ we found that they maintained even higher levels of cognitive performance across all domains compared with the total sample of centenarians. While this group presented a similar rate of decline in memory compared with the total group, we also observed a slight decline in attention/processing speed. This is in agreement with the age-related cognitive decline observed in younger populations, which includes not only a decline in memory but also a decline in executive functions and processing speed.^6,7,8,9,11^ It is possible that having a very high cognitive performance at study inclusion renders an individual more vulnerable for age-related decline thereafter. However, these results should be interpreted with caution: the decline was observed in a reduced sample around the third year after study inclusion, such that we cannot exclude the influence of a potential terminal drop.^54^

Our findings suggest that after reaching age 100 years, cognitive performance remains relatively stable during ensuing years. Therefore, these centenarians might be resilient or resistant against different risk factors of cognitive decline. Evidence for resistance would be supported by low loads or absence of risk factors, while evidence for resilience would be supported by the exposure to such factors in combination with a higher cognitive performance and/or lower rates of decline.^55,56,57^

In the current study, carrying an *APOE* ε4 or an *APOE* ε2 allele was not significantly associated with the performance in any cognitive domain nor with the rate of decline. This may suggest that the effects of *APOE* alleles are exerted before the age of 100 years. This is in line with reports in prospective population studies that the fraction of *APOE* ε4 allele carriers progressively decreases in 80- and 90-year-olds.^34,58^ These ages represent the median age at death in most populations, such that the *APOE* alleles may exert its strong effects on selection during these ages. This is in line with our previous work, in which we found that 19% of centenarians who maintained an MMSE score of 26 or more during at least 2 years follow up carried at least 1 *APOE* ε4 allele compared with 6% of the centenarians who had a lower MMSE at baseline or who declined during follow up.^52^ Together, our findings lead us to speculate that surviving to these extreme ages with an *APOE* ε4 allele implies being resilient against its strong risk-increasing effect.

The postmortem levels of Aβ, NFTs, and NPs in centenarian brains varied widely, but was not associated with cognitive performance or with the rate of decline, which corresponds with our previous findings^42^ and results from the 90+ Study.^59^ This suggests that those centenarians with high neuropathology loads may be resilient against the effects thereof. It is intriguing that the highest stages of Aβ pathology were present in the brains of high performing centenarians, while the highest tau (ie, NFT) and NP levels were not. This indicates that maintained cognitive health may be explained by resilience to the effects of even the highest levels of Aβ pathology, and a combination of resilience and resistance against NFTs and NPs.

Resilience may be further explained by the build-up of cognitive reserve.^15,56^ This concept relates to having more neural resources available by inheritance or lifetime training, allowing higher levels of brain damage to accumulate before clinical symptoms appear.^15^ We found that next to physical health factors, factors of cognitive reserve such as education, frequency of cognitive activity, and premorbid IQ were associated with cognitive performance. This is in line with our previous study,^17^ in which we demonstrated that the cognitively healthy centenarians in our cohort had higher levels of education and a higher socioeconomic background compared with birth cohort peers. However, in this study we found no association between factors of cognitive reserve and the rate of cognitive decline, which is in line with a previous study of 75-year olds.^60^ Together, this suggests that while the effect of cognitive reserve on cognition might still endure at extreme ages, we find no evidence that it is associated with subsequent decline.

### Limitations

This study had several limitations. Because of our inclusion criteria of self-reported cognitive health, the 100-plus cohort of centenarians may not be considered a population-representative sample of centenarians. However, because of the association between cognitive performance and survival,^3,52,61,62,63^ our focus on cognitively healthy centenarians allows participants to be observed longer compared with a representative sample, which is a prerequisite to evaluate trajectories of cognitive performance. Also, investigating a subgroup of healthy centenarians enables the exploration of potential underlying factors of preserved cognitive health and resilience.^64^ Still, the follow-up duration of up to 4 years, and a mean follow-up of 1.6 years in this study, may seem short. However, given previous studies that indicate that 25 years of dementia risk in the younger population is compressed into 2 years in centenarians, a 4-year follow-up in this age group may be considered extremely long.

The high mortality and dementia incidence in our research group confronted us with selective attrition and inherent survivor bias. In our previous study, we observed a higher rate of decline and mortality rate in centenarians who dropped out of the study,^52^ indicating a potential terminal drop.^54^ Therefore, the trajectories reported in this study might be an underestimation of the actual rate of cognitive decline. Nevertheless, we were able to address the differential decline between the cognitive domains before dropout.

We did not present parallel versions of the tests during follow-up visits, therefore we cannot exclude that practice effects may have confounded the assessments of the cognitive trajectories.^65^ Overall, the tests we used to assess cognitive functioning were not designed to measure cognitive decline at extreme ages, and for some tests suitability may be questioned. Indeed, centenarians were not always able to complete tests because of sensory problems, fatigue, or having difficulty understanding instructions.^20^

The limited cognitive decline we observed across domains may have prevented the detection of risk factors associated with cognitive decline after the age of 100 years such that replication of our findings in a study with a large sample of centenarians is warranted. However, note that our findings may be vulnerable to period effects as supported by the higher overall cognitive performance of the large cohort of 95-year olds born in 1915 compared with a large cohort of 93-year olds born in 1905.^66^ Also, our findings may be vulnerable to population effects, as individuals with different population backgrounds (even though they all lived in the same area during the same period) were associated with different dementia incidences.^67^

## Conclusions

In this cohort study, cognitively healthy centenarians were able to maintain their level of cognitive functioning in all investigated cognitive domains with a slight decline in memory function, despite the presence of AD-associated neuropathology and despite being exposed to risk factors of cognitive decline. This provides evidence that some centenarians might be resilient to the effects of neuropathologies and risk factors of cognitive decline.

## Notes

Supplement. **eAppendix.** Multiple Imputation and Creation of Composite Scores **eTable 1.** Overview of Mean Test Scores and Number of Tests Available at Each Time Point **eTable 2.** Latent Class Estimates for Each Cognitive Trajectory **eTable 3.** Linear Mixed Model Regression Coefficients to Investigate Interaction Effects Between Risk Factors and Cognitive Trajectories **eFigure 1.** Flowchart of Inclusion and Number of Participants During Follow-up **eFigure 2.** Comparison Between Groups of Centenarians Who Dropped Out of the Study After 1, 2, 3, or ≥4 Years of Follow-up **eReferences**

## References

[1] Poon LW, Woodard JL, Stephen Miller L, Understanding dementia prevalence among centenarians. J Gerontol A Biol Sci Med Sci. 2012;67(4):358-365. doi:10.1093/gerona/glr250 22389466 PMC3309877

[2] Perls T Dementia-free centenarians. Exp Gerontol. 2004;39(11-12):1587-1593. doi:10.1016/j.exger.2004.08.015 15582273

[3] Mossakowska M, Broczek K, Wieczorowska-Tobis K, Cognitive performance and functional status are the major factors predicting survival of centenarians in Poland. J Gerontol A Biol Sci Med Sci. 2014;69(10):1269-1275. doi:10.1093/gerona/glu003 24509978

[4] Andersen-Ranberg K, Vasegaard L, Jeune B Dementia is not inevitable: a population-based study of Danish centenarians. J Gerontol B Psychol Sci Soc Sci. 2001;56(3):152-159. doi:10.1093/geronb/56.3.P152 11316833

[5] Hagberg B, Bauer Alfredson B, Poon LW, Homma A Cognitive functioning in centenarians: a coordinated analysis of results from three countries. J Gerontol B Psychol Sci Soc Sci. 2001;56(3):141-151. doi:10.1093/geronb/56.3.P141 11316832

[6] Small SA, Stern Y, Tang M, Mayeux R Selective decline in memory function among healthy elderly. Neurology. 1999;52(7):1392-1396. doi:10.1212/WNL.52.7.1392 10227623

[7] Hedden T, Gabrieli JD Insights into the ageing mind: a view from cognitive neuroscience. Nat Rev Neurosci. 2004;5(2):87-96. doi:10.1038/nrn1323 14735112

[8] Park DC, Lautenschlager G, Hedden T, Davidson NS, Smith AD, Smith PK Models of visuospatial and verbal memory across the adult life span. Psychol Aging. 2002;17(2):299-320. doi:10.1037/0882-7974.17.2.299 12061414

[9] Christensen H What cognitive changes can be expected with normal ageing? Aust N Z J Psychiatry. 2001;35(6):768-775. doi:10.1046/j.1440-1614.2001.00966.x 11990887

[10] Salthouse TA Neuroanatomical substrates of age-related cognitive decline. Psychol Bull. 2011;137(5):753-784. doi:10.1037/a0023262 21463028 PMC3132227

[11] Salthouse TA Trajectories of normal cognitive aging. Psychol Aging. 2019;34(1):17-24. doi:10.1037/pag0000288 30211596 PMC6367038

[12] Corrada MM, Brookmeyer R, Paganini-Hill A, Berlau D, Kawas CH Dementia incidence continues to increase with age in the oldest old: the 90+ study. Ann Neurol. 2010;67(1):114-121. doi:10.1002/ana.21915 20186856 PMC3385995

[13] Lobo A, Lopez-Anton R, Santabárbara J, Incidence and lifetime risk of dementia and Alzheimer’s disease in a Southern European population. Acta Psychiatr Scand. 2011;124(5):372-383. doi:10.1111/j.1600-0447.2011.01754.x 21848704

[14] Stern Y Cognitive reserve. Neuropsychologia. 2009;47(10):2015-2028. doi:10.1016/j.neuropsychologia.2009.03.004 19467352 PMC2739591

[15] Stern Y, Arenaza-Urquijo EM, Bartrés-Faz D, Whitepaper: defining and investigating cognitive reserve, brain reserve, and brain maintenance. Alzheimer’s & Dementia. 2020;16(9):1305-1311. doi:10.1016/j.jalz.2018.07.219 30222945 PMC6417987

[16] World Medical Association World Medical Association Declaration of Helsinki: ethical principles for medical research involving human subjects. JAMA. 2013;310(20):2191-2194. doi:10.1001/jama.2013.281053 24141714

[17] Holstege H, Beker N, Dijkstra T, The 100-plus Study of cognitively healthy centenarians: rationale, design and cohort description. Eur J Epidemiol. 2018;33(12):1229-1249. doi:10.1007/s10654-018-0451-3 30362018 PMC6290855

[18] Folstein MF, Folstein SE, McHugh PR “Mini-mental state”: a practical method for grading the cognitive state of patients for the clinician. J Psychiatr Res. 1975;12(3):189-198. doi:10.1016/0022-3956(75)90026-6 1202204

[19] van Buuren S, Groothuis-Oudshoorn K mice: multivariate imputation by chained equations in R. J Stat Software. 2011;45(3):1-67. doi:10.18637/jss.v045.i03

[20] Beker N, Sikkes SA, Hulsman M, Schmand B, Scheltens P, Holstege H Neuropsychological test performance of cognitively healthy centenarians: normative data from the Dutch 100-plus Study. J Am Geriatr Soc. 2019;67(4):759-767. doi:10.1111/jgs.15729 30592018 PMC7379661

[21] Wilson B, Cockburn J, Baddeley A. The Rivermead Behavioural Memory Test. Nederlandstalige bewerking. Handleiding. Balen HGG, Groot Zwaaftink AJM , trans. Swets & Zeitling; 1987.

[22] Wilson B, Cockburn J, Baddeley A. The Rivermead Behavioural Memory Test Reading. Thames Valley Test Co; 1985.

[23] Reitan RM Validity of the Trail Making Test as an indicator of organic brain damage. Perceptual and Motor Skills. 1958;8(3):271-276. doi:10.2466/pms.1958.8.3.271

[24] Wilson BA, Alderman N, Burgess PW, Emslie H, Evans JJ BADS: Behavioural Assessment of the Dysexecutive Syndrome. Pearson; 1996.

[25] Wechsler D. WAIS-III: Wechsler Adult Intelligence Scale. Psychological Corporation; 1997.

[26] Benton AL, Hamsher KD, Sivan A Multilingual Aphasia Examination. Vol 59 AJA Associates; 1989.

[27] Van der Elst W, Van Boxtel MP, Van Breukelen GJ, Jolles J Normative data for the animal, profession and letter m naming verbal fluency tests for Dutch speaking participants and the effects of age, education, and sex. J Int Neuropsychol Soc. 2006;12(1):80-89. doi:10.1017/S1355617706060115 16433947

[28] Warrington EK, James M The Visual Object and Space Perception Battery: VOSP. Pearson London; 1991.

[29] Munang L, Chan M, Lim W Diagnostic performance of the clock drawing test using a pre-drawn circle in persons with early dementia. Asian J Gerontol Geriatr. 2010;5:54-61.

[30] Shulman KI Clock-drawing: is it the ideal cognitive screening test? Int J Geriatr Psychiatry. 2000;15(6):548-561. doi:10.1002/1099-1166(200006)15:6<548::AID-GPS242>3.0.CO;2-U 10861923

[31] Schiepers OJ, Harris SE, Gow AJ, APOE E4 status predicts age-related cognitive decline in the ninth decade: longitudinal follow-up of the Lothian Birth Cohort 1921. Mol Psychiatry. 2012;17(3):315-324. doi:10.1038/mp.2010.137 21263443

[32] Genin E, Hannequin D, Wallon D, APOE and Alzheimer disease: a major gene with semi-dominant inheritance. Mol Psychiatry. 2011;16(9):903-907. doi:10.1038/mp.2011.52 21556001 PMC3162068

[33] Farrer LA, Cupples LA, Haines JL, ; APOE and Alzheimer Disease Meta Analysis Consortium Effects of age, sex, and ethnicity on the association between apolipoprotein E genotype and Alzheimer disease—a meta-analysis. JAMA. 1997;278(16):1349-1356. doi:10.1001/jama.1997.03550160069041 9343467

[34] van der Lee SJ, Wolters FJ, Ikram MK, The effect of APOE and other common genetic variants on the onset of Alzheimer’s disease and dementia: a community-based cohort study. Lancet Neurol. 2018;17(5):434-444. doi:10.1016/S1474-4422(18)30053-X 29555425

[35] Mahoney FI, Barthel DW Functional Evaluation: The Barthel Index. Mary State Matiland GD; 1965. 14258950

[36] Reijnierse EM, de Jong N, Trappenburg MC, Assessment of maximal handgrip strength: how many attempts are needed? J Cachexia Sarcopenia Muscle. 2017;8(3):466-474. doi:10.1002/jcsm.12181 28150387 PMC5476859

[37] United Nations Educational, Scientific, and Cultural Organization International standard classification of education or ISCED-1997. Published 1997 Accessed November 19, 2020. https://en.unesco.org/themes/education

[38] Wilson R, Barnes L, Bennett D Assessment of lifetime participation in cognitively stimulating activities. J Clin Exp Neuropsychol. 2003;25(5):634-642. doi:10.1076/jcen.25.5.634.14572 12815501

[39] Nelson HE, O’Connell A Dementia: the estimation of premorbid intelligence levels using the New Adult Reading Test. Cortex. 1978;14(2):234-244. doi:10.1016/S0010-9452(78)80049-5 679704

[40] Schmand B, Bakker D, Saan R, Louman J The Dutch Reading Test for Adults: a measure of premorbid intelligence level. Tijdschr Gerontol Geriatr. 1991;22(1):15-19. 1877068

[41] Schmand B, Lindeboom J, van Harskamp F Dutch Adult Reading Test. Swets & Zeitlinger; 1992.

[42] Ganz AB, Beker N, Hulsman M, ; Netherlands Brain Bank Neuropathology and cognitive performance in self-reported cognitively healthy centenarians. Acta Neuropathol Commun. 2018;6(1):64. doi:10.1186/s40478-018-0558-5 30037350 PMC6055341

[43] Thal DR, Rüb U, Schultz C, Sequence of Abeta-protein deposition in the human medial temporal lobe. J Neuropathol Exp Neurol. 2000;59(8):733-748. doi:10.1093/jnen/59.8.733 10952063

[44] Braak H, Braak E Neuropathological stageing of Alzheimer-related changes. Acta Neuropathol. 1991;82(4):239-259. doi:10.1007/BF00308809 1759558

[45] Braak H, Alafuzoff I, Arzberger T, Kretzschmar H, Del Tredici K Staging of Alzheimer disease-associated neurofibrillary pathology using paraffin sections and immunocytochemistry. Acta Neuropathol. 2006;112(4):389-404. doi:10.1007/s00401-006-0127-z 16906426 PMC3906709

[46] Braak H, Braak E Staging of Alzheimer’s disease-related neurofibrillary changes. Neurobiol Aging. 1995;16(3):271-278. doi:10.1016/0197-4580(95)00021-6 7566337

[47] Mirra SS, Heyman A, McKeel D, The Consortium to Establish a Registry for Alzheimer’s Disease (CERAD)—part II, standardization of the neuropathologic assessment of Alzheimer’s disease. Neurology. 1991;41(4):479-486. doi:10.1212/WNL.41.4.479 2011243

[48] Nylund KL, Asparouhov T, Muthén BO Deciding on the number of classes in latent class analysis and growth mixture modeling: a Monte Carlo simulation study. Structural equation modeling. 2007;14(4):535-569. doi:10.1080/10705511.2014.882690

[49] Proust-Lima C, Philipps V, Liquet B Estimation of extended mixed models using latent classes and latent processes: the R package lcmm. Preprint. Posted online January 24, 2015 Accessed December 4, 2020. arXiv 150300890. https://arxiv.org/pdf/1503.00890.pdf

[50] Bates D. Computational methods for mixed models. Vignette for lme4. Created October 14, 2007 Accessed November 19, 2020. http://btr0xq.rz.uni-bayreuth.de/math/statlib/R/CRAN/doc/vignettes/lme4/Theory.pdf

[51] Wright DB Some Limits Using Random Slope Models to Measure Academic Growth. Frontiers in Education. 2017;2(58). doi:10.3389/feduc.2017.00058

[52] Beker N, Sikkes SAM, Hulsman M, Longitudinal maintenance of cognitive health in centenarians in the 100-plus Study. JAMA Netw Open. 2020;3(2):e200094. doi:10.1001/jamanetworkopen.2020.0094 32101309 PMC7137688

[53] Smits LL, van Harten AC, Pijnenburg YA, Trajectories of cognitive decline in different types of dementia. Psychol Med. 2015;45(5):1051-1059. 25229325 10.1017/S0033291714002153

[54] Berg S. Aging, Behavior, and Terminal Decline. Academic Press; 1996.

[55] Arenaza-Urquijo EM, Vemuri P Resistance vs resilience to Alzheimer disease: clarifying terminology for preclinical studies. Neurology. 2018;90(15):695-703. doi:10.1212/WNL.0000000000005303 29592885 PMC5894932

[56] Arenaza-Urquijo EM, Vemuri P Improving the resistance and resilience framework for aging and dementia studies. Alzheimers Res Ther. 2020;12(1):41. doi:10.1186/s13195-020-00609-2 32290864 PMC7158381

[57] Montine TJ, Cholerton BA, Corrada MM, Concepts for brain aging: resistance, resilience, reserve, and compensation. Alzheimers Res Ther. 2019;11(1):22. doi:10.1186/s13195-019-0479-y 30857563 PMC6410486

[58] Khachaturian AS, Corcoran CD, Mayer LS, Zandi PP, Breitner JCS; Cache County Study Investigators Apolipoprotein E ϵ4 count affects age at onset of Alzheimer disease, but not lifetime susceptibility: the Cache County Study. Arch Gen Psychiatry. 2004;61(5):518-524. doi:10.1001/archpsyc.61.5.518 15123497

[59] Balasubramanian AB, Kawas CH, Peltz CB, Brookmeyer R, Corrada MM Alzheimer disease pathology and longitudinal cognitive performance in the oldest-old with no dementia. Neurology. 2012;79(9):915-921. 22895581 10.1212/WNL.0b013e318266fc77 PMC3425842

[60] Wilson RS, Yu L, Lamar M, Schneider JA, Boyle PA, Bennett DA Education and cognitive reserve in old age. Neurology. 2019;92(10):e1041-e1050. doi:10.1212/WNL.0000000000007036 30728309 PMC6442015

[61] Hagberg B, Samuelsson G. Survival after 100 years of age: a multivariate model of exceptional survival in Swedish Centenarians. J Gerontol. 2008;63(11):1219-1226. doi:10.1093/gerona/63.11.1219 19038837

[62] Poon L, Johnson M, Davey A, Dawson D, Siegler I, Martin P Psychosocial predictors of survival among centenarians. Facts, Res, and Intervent in Geriatr. 2000:77-89. Accessed December 4, 2020. http://pascal-francis.inist.fr/vibad/index.php?action=getRecordDetail&idt=1449384

[63] Samuelsson SM, Alfredson BB, Hagberg B, The Swedish Centenarian Study: a multidisciplinary study of five consecutive cohorts at the age of 100. Int J Aging Hum Dev. 1997;45(3):223-253. doi:10.2190/XKG9-YP7Y-QJTK-BGPG 9438877

[64] Kawas CH, Corrada MM Successful cognitive aging: what the oldest-old can teach us about resistance and resilience. Neurology. 2020;95(8):329-330. doi:10.1212/WNL.0000000000010251 32699142

[65] Calamia M, Markon K, Tranel D Scoring higher the second time around: meta-analyses of practice effects in neuropsychological assessment. Clin Neuropsychol. 2012;26(4):543-570. doi:10.1080/13854046.2012.680913 22540222

[66] Christensen K, Thinggaard M, Oksuzyan A, Physical and cognitive functioning of people older than 90 years: a comparison of two Danish cohorts born 10 years apart. Lancet. 2013;382(9903):1507-1513. doi:10.1016/S0140-6736(13)60777-1 23849796 PMC3818336

[67] Gilsanz P, Corrada MM, Kawas CH, Incidence of dementia after age 90 in a multiracial cohort. Alzheimers Dement. 2019;15(4):497-505. doi:10.1016/j.jalz.2018.12.006 30797730 PMC6497045

