# Supplementary material for: Association of Cognitive Function Trajectories in Centenarians With Postmortem Neuropathology, Physical Health, and Other Risk Factors for Cognitive Decline
Source: JAMA Netw Open. 2021 Jan 15;4(1):e2031654. doi: 10.1001/jamanetworkopen.2020.31654 (PMC7811180; doi:10.1001/jamanetworkopen.2020.31654)
Supplement: Supplement. — eAppendix. Multiple Imputation and Creation of Composite Scores eTable 1. Overview of Mean Test Scores and Number of Tests Available at Each Time Point eTable 2. Latent Class Estimates for Each Cognitive Trajectory eTable 3. Linear Mixed Model Regression Coefficients to Investigate Interaction Effects Between Risk Factors and Cognitive Trajectories eFigure 1. Flowchart of Inclusion and Number of Participants During Follow-up eFigure 2. Comparison Between Groups of Centenarians Who Dropped Out of the Study After 1, 2, 3, or ≥4 Years of Follow-up eReferences [file jamanetwopen-e2031654-s001.pdf]

Supplemental Online Content

Beker N, Ganz A, Hulsman M, et al. Association of cognitive function trajectories in centenarians with postmortem neuropathology, physical health, and other risk factors for cognitive decline. *JAMA Netw Open*. 2021;4(1):e2031654.
doi:10.1001/jamanetworkopen.2020.31654

eAppendix. Multiple Imputation and Creation of Composite Scores

eTable 1. Overview of Mean Test Scores and Number of Tests Available at Each Time Point

eTable 2. Latent Class Estimates for Each Cognitive Trajectory

eTable 3. Linear Mixed Model Regression Coefficients to Investigate Interaction Effects Between Risk Factors and Cognitive Trajectories

eFigure 1. Flowchart of Inclusion and Number of Participants During Follow-up

eFigure 2. Comparison Between Groups of Centenarians Who Dropped Out of the Study After 1, 2, 3, or ≥4 Years of Follow-up

eReferences

This supplemental material has been provided by the authors to give readers additional information about their work.

© 2021 Beker N et al. *JAMA Network Open.*

eAppendix. Multiple Imputation and Creation of Composite Scores

Reason and number of missing tests

Missing data occurred across cognitive tests and questionnaires for several reasons. First, not all participants were presented with the complete test battery because in some cases fatigue, sensory or motor difficulties prevented individuals to take the test, as described previously.[1] Second, specific tests and questionnaires were not presented to all centenarians as our test protocol gradually expanded over the course of the study. The fraction of missing test scores over all time points was 41.7% for the RBMT, 19.0% for the VAT, 59.4% for the TMT B, 37.4% for the Key search test, 20.4% for Digit span backwards, 11.4% for Letter fluency, 13.2% for Animal fluency, 22.5% for the clock drawing test, 50.3% for the Number location, 19.5% for the Digit span forward, and 39.9% for the TMT A. An overview of missing test scores per time point can be found in Table S1.

Imputation for missing data

We imputed missing 1) cognitive test scores and 2) single items on questionnaires using MICE (multiple imputation with chained equations),[2] based on predictive mean matching method. The MICE tool was found to be effective for imputation of missing cognitive test scores.[3] We assumed that tests or items were missing at random at every time point of follow-up. Therefore, we performed multiple imputation for all time points separately on individual test scores or items, and did not impute missing values for participants that were included later or earlier in the study.

Cognitive test scores

For imputing the missing cognitive test scores, we included all other available cognitive test scores in the imputation model The median number of tests (out of 12 total) available was 9, 9, 8, 8, 8.5 for baseline, 1-year, 2-year, 3-year, and 4-year follow-up respectively. Other auxiliary variables included age, sex, education, sensory capacities, MMSE scores, and

© 2021 Beker N et al. *JAMA Network Open.*

possible reasons for missing test scores as described previously (no understanding of instructions, and fatigue and/or motivation problems).[1]

Imputed scores for individual tests from 20 iterations were used to standardize scores by calculating z scores using the mean and standard deviation of baseline scores. Next, we constructed a total score per cognitive domain, by calculating the average z score across domain-associated tests. A global cognition score was operationalized per time-point by calculating an average z score across all cognitive tests administered at that point in time.

Imputation for missing items on the MMSE and questionnaires

To obtain total scores on the Barthel Index, MMSE, and cognitive activity questionnaire, we performed item-level imputation for missing items, provided ≥80% of all items were available.[4,5] If there were more items missing, scores were not included in the analyses. For the item-level imputation we included age, sex, age, education and individual available items as auxiliary variables. Imputed scores from 20 iterations were used to calculate mean total scores on the questionnaires. Missing items on other factors were not imputed, and for analyses including these factors we omitted samples with missing values.

© 2021 Beker N et al. *JAMA Network Open.*

eTable 1. Overview of Mean Test Scores and Number of Tests Available at Each Time Point

Tests	Baseline (n=330)		One year FU (n=155)		Two year FU (n=77)		Three year FU (n=29)		Four year FU (n=8)	
	No.	Mean (SD)	No.	Mean (SD)	No.	Mean (SD)	No.	Mean (SD)	No.	Mean (SD)
RBMT immediate recall	209	8.0 (4.6)	97	9.2 (5.4)	30	8.7 (5.4)	12	8.6 (5.8)	1	8.0 (NA)
RBMT delayed recall	209	4.7 (4.3)	96	5.5 (5.4)	30	5.1 (4.0)	12	5.2 (5.0)	1	4.5 (NA)
VAT	261	8.1 (3.7)	126	8.5 (3.9)	67	7.9 (4.1)	24	8.6 (3.6)	7	9.4 (3.2)
Trail Making Test B	133	325 (191)	64	318 (154)	33	350 (193)	9	292 (95)	4	396 (197)
Key search	209	6.6 (3.5)	99	6.8 (3.3)	46	7.2 (3.4)	16	8.1 (3.8)	5	5.6 (2.7)
Digit span backwards	265	4.5 (1.5)	128	4.9 (1.7)	59	5.0 (1.5)	19	4.8 (1.3)	6	5.5 (0.8)
Animal fluency	286	11.0 (4.5)	139	12.1 (4.7)	64	13.3 (5.1)	25	12.6 (4.5)	6	14.5 (4.8)
Letter fluency	301	23.0 (10.6)	137	25.5 (10.6)	63	26.4 (9.8)	24	27.0 (8.2)	6	32.5 (12.4)
Clock drawing	260	3.3 (1.4)	117	3.5 (1.3)	56	3.5 (1.2)	24	3.9 (1.3)	7	4.4 (1.0)
Number location	177	8.5 (1.9)	84	8.7 (1.6)	26	8.5 (2.3)	10	7.7 (1.6)	1	8.0 (NA)
Trail Making Test A	195	126 (84)	94	113 (73)	49	119 (68)	16	144 (125)	6	160 (85)
Digit span forwards	264	6.9 (1.8)	130	7.1 (1.8)	59	7.5 (1.9)	23	7.3 (1.9)	6	8.0 (1.9)

Score range of the tests are as follows: digit span: 0-16 (both forwards and backwards), RBMT: 0-42 (both immediate and delayed recall), visual association test: 0-12 (trial 1+2), key search: 0-16 (no time limit), number location: 0-10, and clock drawing: 0-5. Trail Making Test scores are time in seconds. Scores for animal and letter fluency represent number of named items in 1 minute. Higher scores indicate better performance.
Abbreviations: RBMT=Rivermead Behavioural Memory Test. VAT=Visual Association Test.

© 2021 Beker N et al. *JAMA Network Open.*

eTable 2. Latent Class Estimates for Each Cognitive Trajectory

	No. classes	No. parameters	loglik	AIC	BIC	% Class 1	% Class 2
Global cognition							
	1	11	-322.5	666.9	708.5	100	
	2	14	-321.4	670.7	723.7	47.4	52.6
Memory							
	1	11	-592.9	1207.8	1249.4	100	
	2	14	-592.9	1213.8	1266.8	65.2	34.8
Executive functions							
	1	11	-433.0	888.0	929.6	100	
	2	14	-433.0	894.0	947.0	26.2	73.8
Verbal fluency							
	1	10	-609.5	1239.0	1276.9	100	
	2	13	-609.5	1245.0	1294.3	60.2	39.8
Visuospatial functions							
	1	10	-529.0	1078.0	1115.9	100	
	2	13	-525.8	1077.6	1126.9	1.2	98.8
Attention and processing speed							
	1	10	-497.4	1014.9	1052.8	100	
	2	13	-497.4	1020.9	1070.1	44.6	55.4

Separate latent class linear mixed models including a random intercept adjusting for sex, age, education, vision, and hearing capacities. A random slope was not included. Models on verbal fluency were only adjusted for hearing capacities. % indicates percentage of centenarians in each class according to probability of class membership.
Abbreviations: AIC, Akaike's Information Criterion. BIC, Bayesian information criterion.

© 2021 Beker N et al. *JAMA Network Open.*

eTable 3. Linear Mixed Model Regression Coefficients to Investigate Interaction Effects Between Risk Factors and Cognitive Trajectories

	Global cognition		Memory	
	β (95% CI)	p	β (95% CI)	p
Interaction effect models				
Age*Time	0.01 (-0.01 – 0.03)	.44	0.03 (-0.01 – 0.06)	.10
Sex*Time	-0.00 (-0.06 – 0.06)	.95	-0.07 (-0.18 – 0.04)	.19
APOE-e4 presence*Time	0.02 (-0.05 – 0.10)	.53	0.02 (-0.11 – 0.15)	.75
APOE-e2 presence*Time	-0.00 (-0.06 – 0.06)	.91	-0.12 (-0.23 – -0.01)	.03
Factors of physical health				
Living situation*Time	-0.01 (-0.07 – 0.04)	.62	-0.02 (-0.11 – 0.08)	.75
Good hearing*Time	-0.07 (-0.12 – -0.02)	.01	-0.08 (-0.18 – 0.01)	.08
Good vision*Time	0.04 (-0.02 – 0.10)	.20	-0.01 (-0.11 – 0.09)	.85
Barthel Index*Time	-0.01 (-0.07 – 0.05)	.65	-0.07 (-0.18 – 0.03)	.18
History of stroke or TIA*Time	0.01 (-0.05 – 0.07)	.76	0.09 (-0.02 – 0.19)	.12
History of hypertension*Time	-0.00 (-0.06 – 0.05)	.88	0.03 (-0.06 – 0.12)	.53
Grip strength*Time	-0.00 (-0.01 – 0.01)	.85	-0.02 (-0.03 – 0.00)	.06
Factors of cognitive reserve				
High education level*Time	-0.05 (-0.10 – 0.01)	.10	-0.07 (-0.16 – 0.03)	.16
Premorbid IQ*Time	-0.00 (-0.00 – 0.00)	.31	-0.00 (-0.00 – 0.00)	.44
Lifetime cognitive activity*Time	-0.00 (-0.00 – 0.00)	.22	-0.00 (-0.00 – 0.00)	.94
Current cognitive activity*Time	-0.01 (-0.02 – -0.00)	.03	-0.02 (-0.03 – -0.00)	.02
AD-associated neuropathologies				
Aß (Thal phase)*Time	-0.02 (-0.09 – 0.04)	.48	-0.06 (-0.18 – 0.05)	.28
NFTs (Braak stage)*Time	-0.07 (-0.23 – 0.10)	.43	0.02 (-0.28 – 0.33)	.88
NPs (CERAD score 1)*Time	-0.05 (-0.26 – 0.16)	.66	-0.18 (-0.57 – 0.20)	.36
NPs (CERAD score 2)*Time	-0.09 (-0.26 – 0.08)	.31	-0.16 (-0.47 – 0.16)	.34

*Significant after correction for multiple testing (listed p values are uncorrected for multiple comparisons). Separate linear mixed models including a random intercept which were adjusted for sex, age, education, vision and hearing capacities. Models on verbal fluency were only adjusted for hearing capacities. A random slope was not included. There was missing data on *APOE-e* allele (n=44), vision (n=8), hearing (n=4), Barthel Index (n=30), stroke/TIA (n=26), hypertension (n=27), grip strength (n=169), premorbid IQ (n=90), lifetime cognitive activity (n=38) and current cognitive activity (n=43). Score ranges are as follows: High education level: ≥ post-secondary non-tertiary education, low education level: ≤ upper secondary education, Barthel Index (0-20, scores ≥15 indicating independence in ADL), lifetime and current cognitive activity (score range 0-100, and 0-25 respectively, higher scores indicate more frequent cognitive activity), Thal phase (score range 0-5), Braak stage (score range 0-VI) and CERAD score (score range 0-3), higher scores indicate higher levels of pathology.
Abbreviations: AD, Alzheimer's disease, TIA, Transient Ischemic Attack, CERAD, Consortium to Establish a Registry for Alzheimer's disease, Aβ, Amyloid Beta, NFTs, Neurofibrillary Tangles, NPs, Neuritic Plaques.

© 2021 Beker N et al. *JAMA Network Open.*

eFigure 1. Flowchart of Inclusion and Number of Participants During Follow-up

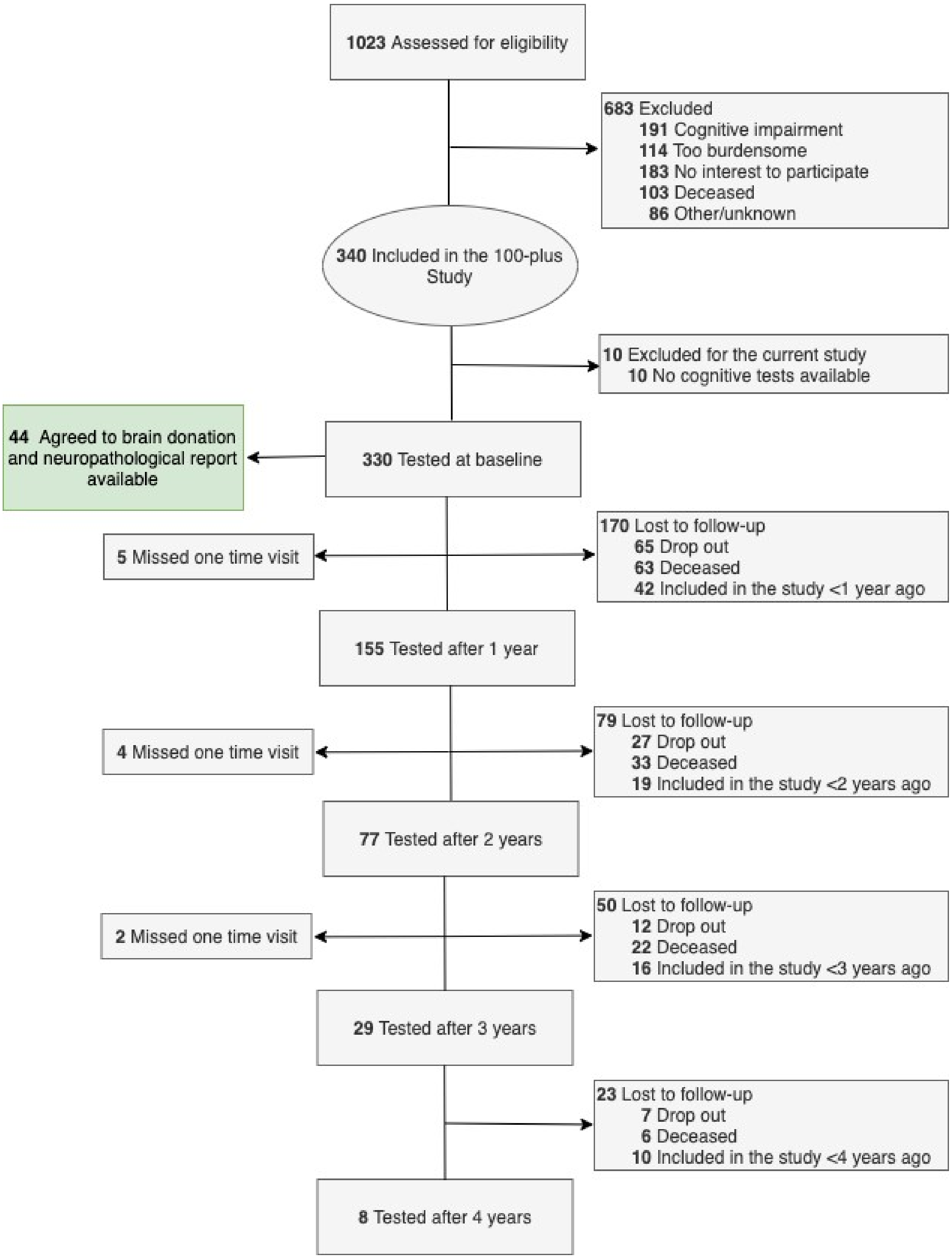

*Some centenarians missed a visit one time, but were visited the year after (n=5, n=4, n=2 for T2, T3, and T4 respectively).

© 2021 Beker N et al. *JAMA Network Open.*

eFigure 2. Comparison Between Groups of Centenarians Who Dropped Out of the Study After 1, 2, 3, or ≥4 Years of Follow-up

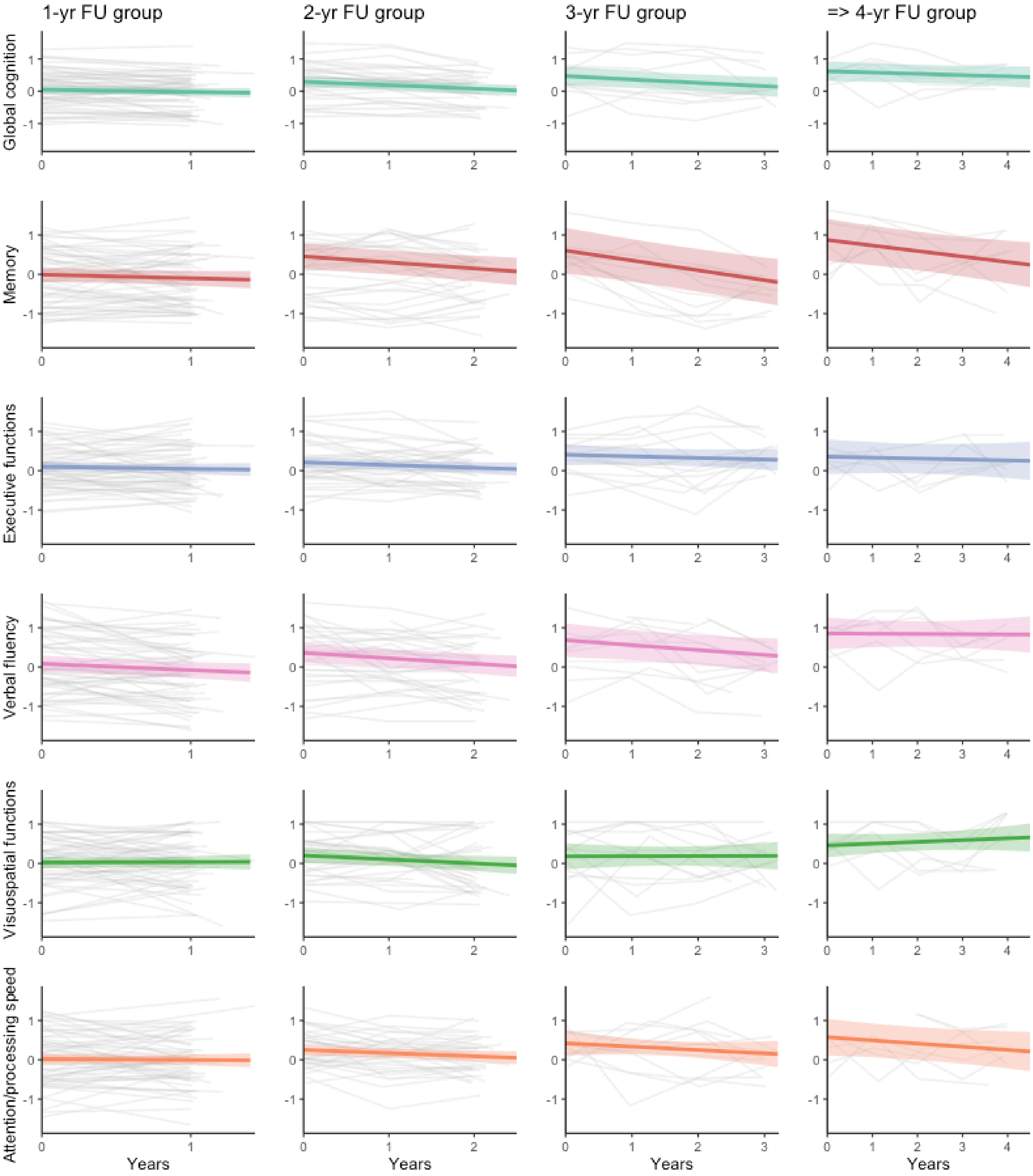

© 2021 Beker N et al. *JAMA Network Open.*

Mean and individual trajectories of cognitive domains based on linear mixed models with random intercept, adjusting for sex, age, and education, hearing and vision capacities. Centenarians still waiting to be visited for follow-up were excluded. The model on verbal fluency was only adjusted for hearing capacities. Gray lines indicate individual trajectories (raw scores), colored lines indicate mean trajectories with time as continuous variable.

© 2021 Beker N et al. *JAMA Network Open.*

eReferences

1. Beker N, Sikkes SA, Hulsman M, Schmand B, Scheltens P, Holstege H. Neuropsychological Test Performance of Cognitively Healthy Centenarians: Normative Data From the Dutch 100-Plus Study. *Journal of the American Geriatrics Society.* 2018.
2. van Buuren S, Groothuis-Oudshoorn K. mice: Multivariate Imputation by Chained Equations in R. *2011.* 2011;45(3):67.
3. Rawlings AM, Sang Y, Sharrett AR, et al. Multiple imputation of cognitive performance as a repeatedly measured outcome. *European journal of epidemiology.* 2017;32(1):55-66.
4. Holstege H, Beker N, Dijkstra T, et al. The 100-plus Study of cognitively healthy centenarians: rationale, design and cohort description. *European Journal of Epidemiology.* 2018.
5. Burns RA, Butterworth P, Kiely KM, et al. Multiple imputation was an efficient method for harmonizing the Mini-Mental State Examination with missing item-level data. *Journal of clinical epidemiology.* 2011;64(7):787-793.

© 2021 Beker N et al. *JAMA Network Open.*
